# Subjective Assessments and Serum Cortisol Levels as Risk Factors of Pain Persistence in the Late Postoperative Period in Old and Oldest-Old Patients

**DOI:** 10.3390/ejihpe13020034

**Published:** 2023-02-15

**Authors:** Vladyslav O. Telegan, Christos Tsagkaris, Sandeep Kumar Singh, Kostiantyn V. Tarasenko

**Affiliations:** 1 Department of Anaesthesiology and Intensive Care, Poltava State Medical University, 36000 Poltava, Ukraine; 2 European Student Think Tank, Public Health and Policy Working Group, 1058 Amsterdam, The Netherlands; 3 Indian Scientific Education and Technology (ISET) Foundation, Lucknow 226002, India

**Keywords:** pain, surgery, cortisol, old age, postoperative outcomes

## Abstract

Postoperative pain is one of the most common postoperative complications, resulting in significant burdens and adverse outcomes among patients, notably the frailest ones. Predicting the likelihood of intense postoperative pain can help optimize a patient’s recovery. The aims of this study were to build a prognostic model of pain persistence in elderly and senile patients in the late postoperative period, based on clinical and laboratory parameters of the early postoperative period, and to evaluate the potential for the model’s application. The study included 201 male and female patients who had undergone surgery of any type from September 2019 to August 2020. The patients were divided into three groups: senile patients, elderly patients, and young patients (control group). All of the examined patients were measured for fasting cortisol on the mornings of the first and seventh days following surgery. A statistically significant increase in the probability of pain persistence in the late postoperative period with the increasing age of the patient was found. Age, pain severity scores on the seventh day after surgery, and cortisol concentration in the blood on the first day after surgery, are of prognostic value for the risk of persistence of postoperative-pain syndrome.

## 1. Introduction

Postoperative pain is one of the most common complaints in surgical practice; hence, its management is of great clinical importance. Evidence suggests that between 35% and 70% of patients who have undergone elective and emergency surgery suffer from postoperative pain. In one of the largest studies of patients in surgical departments in the UK, it was found that 10.9 percent of patients experienced high-intensity postoperative pain, while 29.7 percent of patients experienced medium-intensity pain [1].

According to the International Association for the Study of Pain (IASP), pain is an unpleasant sensation and an emotional experience associated with actual or potential tissue damage or the time described by the patient for such damage [2]. Despite the numerous pharmaceutical and non-pharmaceutical means of analgesia available in the postoperative phase, postoperative pain remains a challenge for both patients and clinicians [3].

The etiology of pain in old and oldest-old patients, who constitute a high-risk demographic for postoperative complications, is complex. Age- and frailty-related alterations in neurohumoral regulation and the emotional burden of nociceptive impulses have a compounding effect on pain-related distress among this group of patients. In particular, the pineal gland undergoes age-related calcification, which causes morpho-functional changes in daily activity and sleep quality [4]; olfactory bulbs and the locus coeruleus area are subjected to hypoxic damage due to chronic cardiovascular diseases [5,6,7], and the cerebral cortex may undergo atrophic changes promoting cognitive and motor decline [8]. Additionally, concurrent somatic illnesses, metabolic disorders, limited functionality and, oftentimes, (perceived) dependence on family members or caregivers contribute to the particularities of pain perception in old and oldest-old patients. The postoperative pain of patients in this age group can diminish the functionality of the patient and elicit stress and depressive symptoms. 

Inadequate management of acute postoperative pain is associated with increased morbidity, functional impairment, reduced quality of life, and increased health care costs. In addition, the existence and intensity of acute pain enable us to predict the likelihood of its chronicity [9]. This risk has been highlighted by both preclinical research and the clinical investigation of laboratory biomarkers in patients in intensive care units, which are aimed at uncovering predictors of the persistence and chronicity of pain in the postoperative period [10]. However, the prediction of the persistence of postoperative pain, based on data from the early postoperative period, is still up for debate.

The aims of this study were to build a prognostic model of pain persistence in old and oldest-old patients in the late postoperative period, based on clinical and laboratory parameters of the early postoperative period, and to evaluate the potential for the model’s application.

## 2. Materials and Methods

The study was conducted in the 3rd Poltava City Hospital from September 2019 to August 2020. 

### 2.1. Participants

The criteria for inclusion in the study were patients aged over 18 years, patients who underwent elective surgery, and patient complaints of postoperative pain of any intensity. Patients younger than 18 years, patients who experienced open surgery, patients receiving chronic pain medication, expectant mothers, patients with delirium in the postoperative period, patients with hormonal and mental disorders in the stages of sub- or decompensation, and patients who underwent neurosurgical operations were excluded from the study, because of the potential impact of the operation on the pain sensation of such patients. A total of 317 patients were evaluated, 116 of whom were excluded from the analysis due to the presence of exclusion criteria (n = 83), the incompleteness of the entire research protocol (n = 21), or the patient’s own request (n = 12). The study included 201 male and female patients who had undergone laparoscopic (n = 164) or robotic (n = 37) surgery. All patients underwent control of perioperative pain in accordance with pertinent guidelines on the control of perioperative pain [11]. Treatment of postoperative pain was carried out by balanced (multimodal) non-opioid analgesia using two or more analgesics that acted by different mechanisms to achieve an optimal analgesic effect without increasing side effects, together with increased doses of one drug. For postoperative analgesia, paracetamol, metamizole, NSAIDs (ketorolac, diclofenac, ketoprofen, dexketoprofen, meloxicam, and celecoxib), and local/regional analgesia were used, according to the intensity of pain.

The examined patients were divided into 3 groups according to age [11]: old patients (65–84 years) were in group 1 (n = 82); oldest-old patients (≥85 years) were in group 2 (n = 21); and young patients (<65 years) were in the control group (n = 98). The sample size and the duration of the study were directed by the available funding and resources at the time. Group 1 included 31 men (31.8%) and 51 women (62.2%); group 2 included 5 men (23.8%) and 16 women (76.2%); the control group included 39 men (39.8%) and 59 women (60.2%). The mean age of patients in group 1 was 66.24 ± 1.82 years; the mean age of patients in group 2 was 87.63 ± 4.21 years; the mean age of patients in control group 3 was 44.59 ± 1.93 years.

### 2.2. Instruments and Measures

To assess the presence and severity of pain, measurements were performed on a 100-point visual analog scale (VAS) on the first and seventh days following surgery (Cronbach’s alpha = 0.84). This scale is based on a 10 cm segment with 100 divisions, where 0 represents no pain and 100 represents unbearable pain. The scale has been previously validated for clinical research in a similar context [12]. The McGill Questionnaire (MPQ) (Cronbach’s alpha = 0.76–0.89), which has three sections—sensory, affective, and evaluative—was used to analyze the perception of postoperative pain. The sensory category describes the different types of pain; the affective category describes how pain affects the psycho-emotional state; the evaluative category indicates the severity of the pain syndrome. The evaluative scale includes one question that describes the subjective perception of pain severity by the patient, where 0 is the absence of pain and 5 is unbearable pain. Utilizing the MPQ, the sensory and affective rank pain index (RPI), which is the sum of the ranks of the selected descriptors, and the index of the number of selected descriptors (INSR), i.e., their number, were calculated. Because patients might be physically or mentally debilitated following surgery and the results might have been skewed by pharmacological effects on the first day, the MPQ was only used on the seventh day [13].

All examined patients were measured for fasting cortisol in the morning on the first and seventh days. The blood sampling procedure was performed according to the general requirements for medical and biological tests at Poltava State Medical University, Ukraine [14]. A nurse took blood from patients’ veins in the immediate presence of a doctor. The concentration of cortisol was determined in the blood serum using a set of reagents for a quantitative enzyme-linked immunosorbent assay, “SteroidIFA-cortisol” (AlcorBio, St. Petersburg, Russia) [15].

### 2.3. Statistical Analysis

Statistical analysis was performed using IBM SPSS Statistics 26.0 (IBM Inc., Armonk, NY, USA). Qualitative variables were presented in the form of absolute numbers and percentages with relative deviation. Χ^2^-Pearson was used to compare qualitative variables. Quantitative data were presented according to the distribution, which was analyzed according to the Kolmogorov–Smirnov criterion. Data that corresponded to the normal distribution were presented as mean (M) and standard error (m) and compared using one-way analysis of variance ANOVA, with a posteriori comparison according to the Scheffe test. To build a prognostic model, we carried out a multivariate binary logistic analysis with the calculation of the coefficients of the prognostic equation and the determination of the odds ratio and a 95% confidence interval for significant factors. Quality assessment of the prognostic model was performed by ROC analysis with the choice of maximum sensitivity and specificity. The choice of the optimal limit value of the test was performed by the method of calculation of the Yoden Index. For all measurements, the critical value was *p* < 0.05, at which the results were considered statistically significant.

### 2.4. Ethics

All patients included in the study provided informed consent in the preoperative period. The study was approved by the Bioethics Committee of Poltava State Medical University and conducted in accordance with the basic provisions of the “Ethical Principles for Medical Research Involving Human Subjects”, approved by the Declaration of Helsinki (1964–2013) and following the recommendations of good clinical practice (ICH GCP) (1996) and orders of the Ministry of Health of Ukraine No. 690, dated 23 September 2009, No. 944, dated 14 December 2009, and No. 616, dated 03 August 2012.

## 3. Results

On the first day following surgery, 11 patients in the first group reported mild pain (13.4 ± 0.68%), 30 patients reported moderate pain (36.6 ± 0.98%), 23 patients reported severe pain (28.0 ± 0, 78%), 15 patients reported extremely severe pain (18.3 ± 0.69%), and three patients reported unbearable pain (3.7 ± 0.11%). Among group 2 patients, there were no patients who described the pain as mild or unbearable. Moderate pain was detected in eight patients of group 2 (38.9 ± 0.34%), severe pain was detected in nine patients of group 2 (49.2 ± 0.85%), and very severe pain was detected in four patients of group 2 (19.0 ± 0.08%). Among patients in the control group, 13 (13.3 ± 0.17%) did not indicate the intensity of pain, 34 (34.7 ± 0.44%) reported mild pain, 29 (29.6 ± 0.39%) reported moderate pain, 13 reported severe pain (13.3 ± 0.87%), eight reported very severe pain (8.2 ± 0.09%), and one reported unbearable pain (1.0 ± 0.02%). 

Seven days after surgery, in group 1, mild pain was noted in 16 patients (19.5 ± 0.65%), moderate pain was noted in 21 patients (25.6 ± 0.53%), severe pain was noted in 19 patients (23.2 ± 0, 78%), and very severe pain was noted in 14 patients (17.1 ± 0.43%). In group 2, mild pain was noted in one patient (4.8 ± 0.16%), moderate pain was noted in 3 patients (14.3 ± 0.28%), severe pain was noted in 13 patients (61.9 ± 0.71%), and very severe pain was noted in two patients (9.5 ± 0.13%). In the control group, mild pain was noted in 24 patients (24.5 ± 0.59%), moderate pain was noted in 25 patients (25.5 ± 0.93%), severe pain was noted in 14 patients (14.3 ± 0.45%), and very severe pain was noted in seven patients (7.1 ± 0.14) %). 

**Figure 1:**
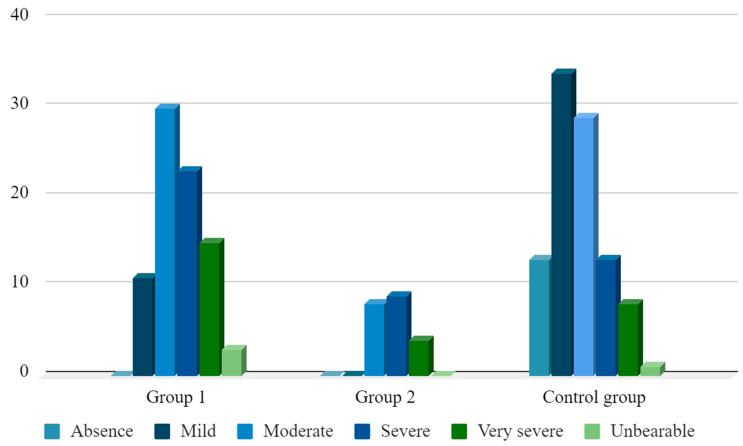
The severity of postoperative pain on the evaluative component of the McGill scale (MPQ) for patients of different ages.

On the seventh day, the subjective perception of postoperative pain was detailed according to the MPQ. According to the MPQ evaluation scale, patients in groups 1 and 2 indicated moderate postoperative pain (2.62 ± 0.12 points and 2.81 ± 0.16 points, respectively), while in the control group a slight degree of pain intensity was determined (1.71 ± 0.12 points). The distribution of postoperative pain levels by the evaluative component of the McGill scale were analyzed according to the groups [Table 1, Figure 1].

It was found that more intense pain is characteristic for older people in the early postoperative period on the first day (χ^2^ = 43.49, *p* < 0.001) and on the seventh day (χ^2^ = 30.98, *p* < 0.001).

Significant differences were found between group 1 and the control group (*p* < 0.001) and between group 2 and the control group (*p* < 0.001). In addition, the evaluative assessment of old patients did not differ in a statistically significant way from that of oldest-old patients (*p* = 0.772). 

The RPI on the MPQ sensory scale in group 1 was 9.27 ± 0.62 points; in group 2, it was 11.91 ± 1.09 points; in the control group, it was 4.75 ± 0.28 points [Table 1]. The INSD on the MPQ sensory scale in group 1 was at the level of 13.77 ± 1.06 points; in group 2, it was 30.86 ± 3.39 points; in the control group, it was 6.05 ± 0.37 points.

Both parameters of the sensory scale showed statistically significant differences among the groups (F = 36.34, *p* < 0.001 for the RPI and F = 81.76, *p* < 0.001 for the INSD). In group 1, the RPI on the sensory scale was higher than that of the control group (*p* < 0.001) and lower than that of group 2 (*p* = 0.045), while the value in group 2 was higher than that of the control group (*p* < 0.001). The INSD on the sensory scale in the control group was lower than that of group 1 (*p* < 0.001) and group 2 (*p* < 0.001), and the INSD was lower in group 1 than in group 2 (*p* < 0.001).

According to the affective scale, the RPI in group 1 was 5.32 ± 0.39 points; in group 2, it was 4.91 ± 0.73 points; in the control group, it was 1.99 ± 0.14 points. The differences among the groups were statistically significant (F = 37.87, *p* < 0.001). In the control group, this indicator was lower than it was in group 1 (*p* < 0.001) and group 2 (*p* < 0.001). However, no statistically significant difference was found between the old and oldest-old groups (*p* = 0.968). The INSD on the affective scale in group 1 was at the level of 6.40 ± 0.44 points; in group 2, it was 11.52 ± 1.55 points; in the control group, it was 6.23 ± 0.42 points. The indicators had statistically significant differences among the groups (F = 12.77, *p* < 0.001). In particular, in group 2, the INSD on the affective scale was higher than it was in group 1 (*p* < 0.001) and the control group (*p* < 0.001). However, no statistically significant difference was found between group 2 and the control group (*p* = 0.892).

The level of cortisol in old patients on the first postoperative day was 649.82 ± 4.62 nmol/L; in oldest-old patients, it was 661.42 ± 7.81 nmol/L. These levels did not vary, statistically, from one another (*p* = 0.967). In addition, in patients in the control group, this figure was significantly lower (*p* < 0.001), at 589.99 ± 10.27 nmol/L. After 7 days in group 1, the level of cortisol decreased to 536.46 ± 3.82 nmol/L; in group 2, it decreased to 521.37 ± 9.58 nmol/L; in the control group, it reduced to 531.39 ± 3.65 nmol/L. These decreases were not statistically different (*p* = 0.223) (Table 2).

On the 14th day after surgery in the late postoperative period, 67 cases of postoperative pain were identified, of which 34 (41.5%) were in group 1, 16 (76.2%) were in group 2, and 17 (17.3%) were in the control group. A statistically significant increase in the probability of pain persistence in the late postoperative period with the increasing age of the patient was found (χ^2^ = 31.07, *p* < 0.001).

To build a prognostic model, a multivariate binary logistic regression analysis of clinical and psychophysiological predictors of postoperative-pain persistence was performed by the Wald reverse inclusion method, with a threshold for inclusion of *p* < 0.05, and a threshold for exclusion of *p* < 0.1. The model is summarized in seven steps, while the latter has four independent predictors (Table 3).

We determined that age, the score on the VAS scale, the score on the MPQ evaluation scale on the seventh day, and the cortisol concentration in the blood on the first day after surgery are of prognostic value for the risk of persistence of postoperative-pain syndrome. The chance of persistence of postoperative pain increases 1.08 times (OR = 1.08, 95% CI 1.02–1.15, *p* = 0.009) for each year of higher age, 1.26 times (OR = 1.26, 95% CI 1.13–1.41, *p* < 0.001) for each point on a 100-point VAS scale on the seventh day, 11.37 times (OR = 11.37, 95% CI 1.45–88.96, *p* = 0.021) for each point on the evaluation MPQ scale, and 2.36 times (OR = 2.36, 95% CI 1.07–4.99, *p* = 0.013) for each nmol/l of cortisol concentration on the first day after surgery.

Thus, the risk factors for persistence of postoperative-pain syndrome are the patient’s age, the assessment of pain severity on the VAS scale, the assessment of the evaluation MPQ scale on the seventh day, and the concentration of cortisol in blood on the first day.

Using the multivariate binary logistic regression analysis, we built a prognostic model of the risk of postoperative-pain persistence, which is described by Equation (1):

p = 1/(1 + exp(10.01–0.78 ∗ X1–0.23 ∗ X2–2.43 ∗ X3–0.86 ∗ X4))),(1)

where

- X1—age, years;
- X2—VAS scale on the seventh day, in points;
- X3—evaluation MPQ scale, in points;
- X4—concentration of cortisol in blood on the first day, in nmol/liter.

Classification characteristics of the constructed model for predicting the risk of persistence of postoperative pain are presented in Table 3.

The accuracy of the constructed prognostic model was evaluated according to the data of the training sample and was 92.5% (95% CI 88.2–96.8%). Point and interval estimates of the efficiency of the regression model were calculated according to the matrix of classifications and were:

- the probability of a correctly predicted positive result (sensitivity) when using this model—87.1% (95% CI 82.9–91.3%);
- the probability of a correctly predicted negative result (specificity)—95.4% (95% CI 93.7–97.1%);
- the predictability of a positive test result—91.0% (95% CI 85.9–96.1%).
- the predictability of a negative test result—93.3% (95% CI 86.9–99.7%).

**Figure 2:**
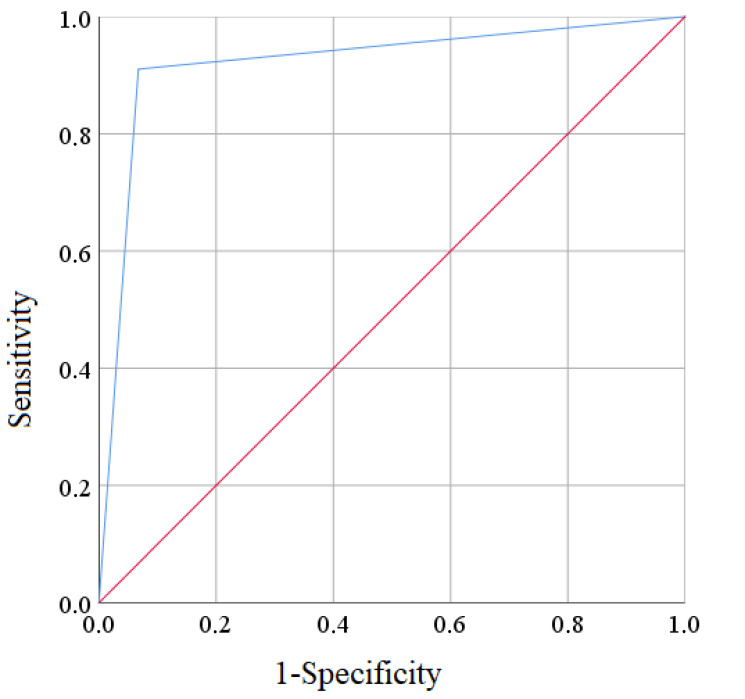
ROC curve of the prognostic model of postoperative-pain persistence in the old and oldest-old patients.

To determine the adequacy of the constructed model, we analyzed the curve of the operational characteristics, which corresponded to our regression equation. See Figure 2. 

The constructed four-factor model revealed the dependence of the risk of postoperative-pain persistence on factor indicators; the area under the ROC curve AUC = 0.92 ± 0.02 (95% CI 0.88–0.97) was statistically significant (*p* < 0.001) and exceeded 0.5, which was evidence of the adequacy of the constructed model (Figure 2).

## 4. Discussion

We found that old and oldest-old patients experience moderate postoperative pain, while young people tend to report pain of low intensity. In advanced age, the sensory component of pain assessment becomes more severe. This is true for both the intensity and variety of pain sensations, and it is more prominent in oldest-old and old patients than in younger and healthier individuals. Subjectively, higher-age patients report that postoperative pain is physically more pronounced, but the range of experiences caused by pain is greater in oldest-old age patients than in old and young patients. In the old and oldest-old patients, the concentration of cortisol in the blood is generally higher than that of young people; this higher concentration can cause increased stress levels and, subsequently, stress-mediated responses to surgery [16]. 

The literature on patients’ pain experiences remains controversial. Van Dijk et al. (2021) reported a mild, and potentially not clinically significant, increase in postoperative pain as a function of increasing age, based on a cohort of 11,510 patients from 26 countries [17]. Younger patients undergoing lumbar spine surgery seem to experience more pain than their older counterparts. However, the reported difference are statistically significant rather than clinically significant [18]. Postoperative pain among patients undergoing robotic surgery is reportedly of lower intensity and shorter duration compared to postoperative pain associated with open or laparoscopic surgery [19,20]. Nevertheless, the differences in postoperative pain among patients of different age groups undergoing robotic surgery are not discussed in depth in relevant recent studies, which otherwise point out a reduction of postoperative pain following robotic surgery [21]. Related scholarship has also highlighted the cofounding role of sex and preoperative pain on the reported levels of postoperative pain [15]. Therefore, the age differences in pain following robotic surgery needs to be more thoroughly studied among different age groups undergoing this type of surgery. Taking into account modifiable and non-modifiable preoperative cofounders could help shed more light on the problem [15]. 

To date, various models for assessing the course of the postoperative period have been proposed and used [16,17]. Our model focused on postoperative pain, which is a common and quite burdensome aspect of surgical care. The median direct additional cost per patient attributed to acute postoperative pain has been estimated at USD 9.46 [19]. It is reasonable to assume that the cumulative direct and indirect costs (out-of-pocket payment, delayed return to everyday activity, need for formal and informal care) pose a challenge for healthcare insurance providers, patients, and their families. In the long term, and in the case of progression to chronic pain, costs can rise to an avalanche, given that persistent postoperative pain can increase the costs of care by up to 40% [19]. Identifying risk factors of postoperative pain can help reduce the physical, mental, and social burdens of the condition and the subsequent financial implications. New approaches to postoperative-pain evaluation need to be effective and, simultaneously, feasible. Our proposed model allows us to predict the risk of postoperative-pain persistence by clinical and laboratory parameters that can be easily assessed in the early postoperative period.

Our study is subject to a number of limitations. Due to limited funds, the sample size and the follow-up period could not be increased further than reported. Collecting relevant data from multiple institutions at national or international levels can indicate whether our results are repeatable. Additional variables related to the type of surgical interventions—including the duration of anesthesia, the pharmacological groups of drugs, and patients’ mental status, which may impact on perceived pain severity, have not been analyzed. Presently, we focused on the evaluation of postoperative-pain differences among three specific age and morbidity groups. Based on our findings, it is possible to conduct further subgroup analysis and to unravel the predictors of pain intensity among old and oldest-old patients.

## 5. Conclusions

In this article, we were able to predict the persistence of pain in the late postoperative period by identifying clinical, psychophysiological, and laboratory characteristics of the early postoperative period. Age, pain severity on the VAS scale and the MPQ evaluation scale on the seventh day, and cortisol concentration in the blood on the first day are the four characteristics we used to build a binary logistic regression model to predict the risk of pain-syndrome persistence in the late postoperative period.

## Figures and Tables

**Table 1:** Features of the assessment of postoperative-pain syndrome on the McGill scale (MPQ) for patients of different ages.

MPQ Indicator	Group			*p*-Value
	Group 1 (*n* = 82)	Group 2 (*n* = 21)	Control Group (*n* = 98)	
Evaluative scale	2.62 ± 0.12 *	2.81 ± 0.16 *	1.71 ± 0.12	<0.001 (1:3–<0.001; 2:3–<0.001; 1:2–0.772)
RPI on sensory scale	9.27 ± 0.62 */**	11.91 ± 1.09 *	4.75 ± 0.28	<0.001 (1:3–<0.001; 2:3–<0.001; 1:2–0.045)
INSD on sensory scale	13.77 ± 1.06 */**	30.86 ± 3.39 *	6.05 ± 0.37	<0.001 (1:3–<0.001; 2:3–<0.001; 1:2–<0.001)
RPI on affective scale	5.32 ± 0.39 *	4.91 ± 0.73 *	1.99 ± 0.14	<0.001 (1:3–<0.001; 2:3–<0.001; 1:2–0.968)
INSD on affective scale	6.40 ± 0.44 **	11.52 ± 1.55 *	6.23 ± 0.42	<0.001 (1:3–0.892; 2:3–<0.001; 1:2–<0.001)

Note: *—statistically significant differences relative to the control group according to the results of ANOVA with Bonferoni correction; **—statistically significant differences relative to group 2 according to ANOVA with Bonferoni correction.

**Table 2:** The results of multivariate logistic regression analysis of laboratory markers of persistence of postoperative pain.

Parameter	Coefficient B ± m	Odds Ratio	95% Confidence Index	*p*-Value
Constant	−10.01 ± 4.66	-	-	0.031 *
Age	0.78 ± 0.03	1.08	1.02–1.15	0.009 *
VAS, day	0.23 ± 0.06	1.26	1.13–1.41	<0.001 *
Evaluative MPQ scale	2.43 ± 1.05	11.37	1.45–88.96	0.021 *
Cortisol, the first day	0.86 ± 0.08	2.36	1.07–4.99	0.013 *

Note: *—statistically significant parameters according to regression analysis.

**Table 3:** The matrix of classifications of the constructed model for determining the risk of postoperative-pain persistence.

Cases That Have Been Observed	Predicted Cases		Total
	Persistence of Pain Was Predicted	No Pain Persistence Was Predicted	
Patients with persistent postoperative pain	61	6	67
Patients without persistent postoperative pain	9	125	134
Total	70	131	201

## Data Availability

Data have been stored by the Department of Anesthesiology in the Poltava State Medical University. They are available upon request.
